# Onto the differences in formulating micro-/nanoparticulate drug delivery system from Thai silk and Vietnamese silk: A critical comparison

**DOI:** 10.1016/j.heliyon.2023.e16966

**Published:** 2023-06-02

**Authors:** Ngoc Yen Nguyen, Thi Ngoc Phuong Nguyen, Nguyen Ngoc Huyen, Van De Tran, Tran Thi Bich Quyen, Huynh Vu Thanh Luong, Duy Toan Pham

**Affiliations:** a Faculty of Chemical Engineering, College of Engineering, Can Tho University, Can Tho 900000, Viet Nam; b Faculty of Medical Sciences, Dong Nai Technology University, Dong Nai 76000, Viet Nam; c Faculty of Public Health, Can Tho University of Medicine and Pharmacy, Can Tho 900000, Viet Nam; d Department of Health Organization and Management, Can Tho University of Medicine and Pharmacy, Can Tho 900000, Viet Nam; e Department of Chemistry, College of Natural Sciences, Can Tho University, Can Tho 900000, Viet Nam

**Keywords:** Silk, Fibroin, Thai, Vietnamese, Nanoparticles, Comparison

## Abstract

Silk fibroin is a natural polymer with physicochemical properties heavily dependent on its silkworm sources and cultivation conditions. Hence, this study critically compared the characteristics and capacity to generate micro-/nanoparticles of fibroin extracted from the Thai silk and Vietnamese silk. Both Thai fibroin (SFT) and Vietnamese fibroin (SFV) were extracted and fabricated into micro-/nanoparticles using the same methods of desalination and condensation, respectively. Firstly, the amino acid compositions of SFT and SFV were determined and found to be similar, suggesting that the different cultivation conditions did not alter the fibroin chemical contents. Secondly, utilizing various analytical techniques, the SFT structure revealed less heavy chains, more light chains and P-25 glycoproteins, and lower crystallinity than those of SFV. Accordingly, compared to the particles formed by SFT, the SFV-based particles were significantly bigger (∼1700 nm vs. ∼150 nm), and possessed less drug (Amphotericin B) entrapment efficiency (64.3 ± 4.4% vs. 79.3 ± 5.1%), higher hemototoxicity, and less biostability in the blood. Conclusively, these differences add more insights for the appropriate applications of each fibroin kind to best promote its qualities and effectiveness.

## Graphical abstract

**Figure:**
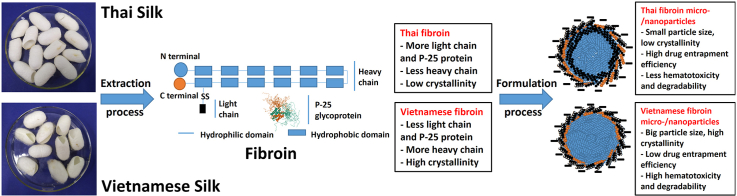

## 1 Introduction

The primary forms of silk are produced by the *Bombyx mori* L. silkworm species, which was discovered and exploited around 4000 years ago [1]. Silk fibers are generated by the silkworm's rear gland and form cocoons in the late pupal stages before full pupation. The filaments are securely connected by a glue-like sericin layer over the fibroin core, with a fibroin core and a sericin shell ratio of 75:25 w/w [2]. Generally, fibroin, a main protein derived from raw silk fibers, composes of 5507 amino acids, of which the majority are glycine (43%), alanine (30%), and serine (12%), which form two polypeptide chains, the heavy chains and the light chains, connected in a 6:6:1 ratio by a P25-glycoprotein [[3], [4], [5]]. The heavy chain is separated into 12 hydrophobic segments interlinked by 11 hydrophilic chains. The hydrophobic segments' repetitive sequences of glycine and alanine, together with other amino acids such as serine, tyrosine, valine, and threonine form the crystalline anti-parallel β-sheet structure via intramolecular and/or intermolecular interactions [6,7]. For light chains, amino acids namely glutamic acid, aspartic acid, arginine, and lysine are placed non-sequentially, forming a semi-crystalline area in the fibroin structure [8]. Although the fibroin structure and molecular/chemical compositions have been well defined, its polymorph and crystallinity index (i.e., the fractions/ratios of the crystalline regions of the fibroin molecule) are strongly affected by the silk sources, extraction methods, formulation platforms, and fabrication techniques [[9], [10], [11], [12]]. These differences in fibroin polymorph consequently result in numerous alteration in its physico-chemical properties such as stability, degradability, drug entrapment and loading efficiency, drug release rates, and cell-material interactions [[3], [4], [5],^8^].

Fibroin, due to its natural biodegradability, biocompatibility, and structure self-modifiability, has been utilized in various drug delivery systems namely hydrogels, films, microparticles, and nanoparticles, for the administration by oral, parenteral, transdermal, and ophthalmic routes [1,10,13,14]. Among them, the micro-/nanoparticulate systems are increasingly gaining attention [2]. However, these systems’ properties are heavily dependent on fibroin crystallinity. For instance, the crosslinked fibroin nanoparticles (FNP) with higher crystallinity possess higher drug entrapment efficiency, lower entrapped drug solubility, and longer drug release profiles, compared with the FNP with lower crystallinity [8]. Additionally, the high-crystallized particles had better stabilities in the blood and more enhanced cellular internalizations than the low-crystallized ones [4].

The *Bombyx mori* L. domesticated silkworm species, like other animals, generate varying silk quality under different growth circumstances, which influences fibroin characteristics. The nutrition of mulberry leaves (*Morus* sp.*,* Moraceae), the main feed of *Bombyx mori* L., and the living environment such as temperature, humidity, ventilation, and light, have significant impacts on the growth and development of silkworm larvae and cocoons [15]. Typically, a silkworm life cycle has five phases of (1) egg stage, (2) silkworm stage, (3) cocoon stage, (4) pupa stage, and (5) butterfly stage [15,16]. Firstly, each female may lay 400–500 eggs, which grow into full embryos in 10–12 days before larvae emerge; this stage is heavily influenced by temperature [15]. Secondly, the silkworm will expand to prepare for the cocooning stage, at which point the meal nutrients are critical [17,18]. Lastly, the period of silkworm larvae and cocoons lasts 21–25 days, following which the silkworm pupates and transforms into a butterfly in 10–15 days. At these stages, the living environment has strong effects [19]. In Thailand, the farming profession silkworms are common in the northeast areas, which have a tropical weather with an average temperature of 28.6 °C, a lengthy period of warm weather followed by cold weather in winter. On the other hand, silkworm farming thrives in Vietnam's Central and Northern areas, which have a humid subtropical environment with an average annual temperature of more than 21 °C and four distinct seasons, making the weather excellent for agricultural development. Mulberry cultivation is also highly established in general, with a comparatively substantial supply of mulberry leaves. Due to the varied climatic conditions and the agricultural environment between Thailand and Vietnam, the properties of silk and fibroin should be significantly differed between these two countries.

Taken these aforementioned issues into consideration, to assess the impact of raw materials on the drug delivery system properties, this study, for the first time, critically investigated the differences, focused on the fibroin crystallinity and related properties, between the Thai and Vietnamese silks. Representative products made from these fibroins, micro-/nanoparticles employing amphotericin B (AmB) as a model drug, were also compared. AmB was selected because (1) it is a water-insoluble lipophilic drug that commonly requires a carrier such as nanoparticles to effectively deliver it to the sites of actions [9], and (2) it possesses a big molecular structure with numerous hydroxyl groups that could interact with fibroin molecules. The study findings could add more insights on the effects of fibroin sources on its respective drug delivery formulations, and thus, careful considerations should be employed for the appropriate applications of each fibroin types.

## 2 Materials and methods

### 2.1 Materials

Thai and Vietnamese *Bombyx mori* L. silkworm cocoons were employed in this investigation. The Thai dried silkworm cocoons were supplied by Bodin Thai Silk Khorat Co., Ltd, Nakhon Ratchasima, Thailand, and the Vietnamese dried silkworm cocoons were obtained directly from farmers in Truc Ninh, Nam Dinh, Vietnam. Both types of cocoons were selected at the same growth phase (40 days after the eggs hatched), with similar appearances, shapes, and physical properties. Silkworm cocoons were gathered and maintained at room temperature after silkworm pupae were removed. All other storage and delivery conditions were kept comparable between the two silk types. The sheep blood was bought from Nam Khoa Limited Liability Company, Vietnam (the blood was obtained from the slaughter house, kept in specific containers with sodium citrate as an anticoagulant), with ISO 9001, ISO 13485, and WHO GMP standards, and completely used within 01 week. AmB was imported from Sigma-Aldrich, Singapore. The chemicals sodium carbonate (Na_2_CO_3_), calcium chloride (CaCl_2_), calcium nitrate (Ca(NO_3_)_2_), and ethanol 99% were supplied by Xilong, China. The dialysis tubing cellulose membrane (12000 MWCO) was provided by Sigma-Aldrich, Singapore. Other chemicals were of reagent grade or higher.

The utilized instruments were UV–Vis spectrophotometer (Jasco V730, Japan), benchtop centrifuge (Mikro 220R, Hettich, Germany), freeze-dryer (Heto PowerDry LL3000, Thermo Fisher, USA), magnetic stirrers (IKA C-MAG HS10, Germany), infrared spectrometer (NICOLET 6700, Thermo, USA), X-ray diffractometer (D8 Advance, Bruker, USA), differential scanning calorimetry machine (DSC 200 F3, Netzsch, Germany), zeta potential analyzer (Zetasizer Ultra, Malvern Panalytical, UK), and scanning electron microscopy (S4800, Hitachi, Japan).

### 2.2 Silk fibroin extraction

**Fig. 1:**
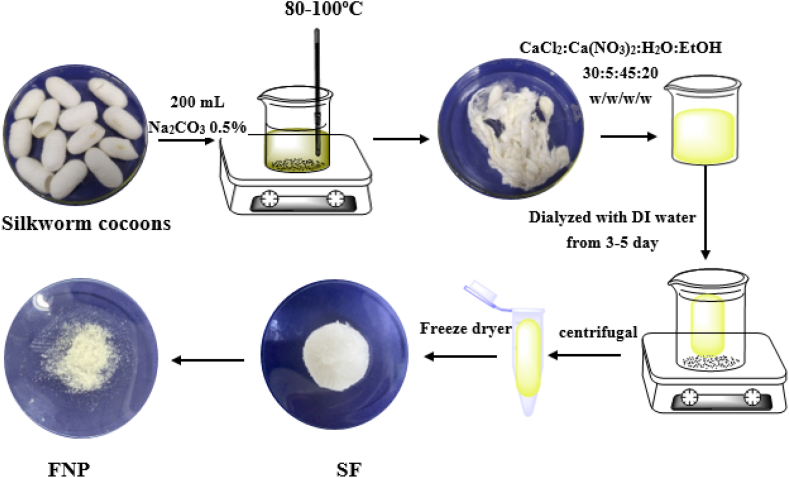
Silk fibroin extraction and fibroin micro-/nanoparticles formulation process from the Thai and Vietnamese silkworm cocoons.

The fibroin was extracted from the Thai (SFT) and Vietnamese (SFV) silk cocoons using the protocol developed by our group [20,21]. To that end, the silk cocoons were degummed to remove sericin by Na_2_CO_3_ 0.5% solution at 100 °C for 1 h, followed by washing thrice with deionized (DI) water and drying at ambient temperature. Then, the degummed silk was dissolved in mixture of CaCl_2_:H_2_O:Ca(NO_3_)_2_:EtOH with weight ratio of 30:45:5:20 at 90 °C to obtain the fibroin solution. Finally, the fibroin solution was dialyzed at room temperature for 3 days, and the product was lyophilized at −40 °C. The silk fibroin was preserved at 4 °C until further uses (Fig. 1).

### 2.3 Fibroin micro-/nanoparticles (FNP) formulations

The FNP from SFT and SFV were prepared by the simple desolvation method [8]. A total of four (04) formulas were fabricated, including the blank FNP-SFT, the blank FNP-SFV, the AmB loaded FNP-SFT, and the AmB loaded FNP-SFV. To make the blank particles, 1.5 mL of ethanol (99%) was added to 0.5 mL of fibroin (SFT or SFV) solution (1% w/v) and the FNP were spontaneously formed. The mixture was stabilized for 24 h, centrifuged at 18000 rpm to separate the product, and the particles were re-dispersed in DI water by sonication, washed thrice with DI water, followed by lyophilization for further use. For the AmB loaded FNP, 0.5 mg of the AmB powder was dissolved in the ethanolic phase and the process was conducted similar to the blank particles.

### 2.4 Silk fibroin characterizations

The characteristics of the SFT and SFV were determined and compared, in terms of physical properties, amino acid compositions, molecular weights, molecular structures, and crystallinity.

#### 2.4.1 Physical properties

The physical properties, in terms of appearance, texture, and stability, of the SFT and SFV were observed and reported as images.

#### 2.4.2 Amino acid compositions

The amino acid compositions of SFT and SFV were determined utilizing the high performance liquid chromatography (HPLC, Agilent, USA) with an automatic sampler, a reverse-phase column XDB C18 (250 mm × 4.6 mm x 5 μm), a fluorescence detector (excitation wavelength of 260 nm and emission wavelength of 320 nm), an injection volume of 10 μL, a flow rate of 1.75 mL/min, and a mobile phase consisted of acetonitrile (A) and 10 mM Na_2_HPO_4_ and 10 mM Na_2_B_4_O_7_ (pH 8.2) (B) with a gradient running mode (0–2.5 min, 15% A; 2.5–19 min, 22% A; 19–31 min, 28% A; 31–45 min, 35% A; 45–48 min, 70% A; and 48–50 min, 15% A). The process was fully validated followed the International Conference on Harmonisation (ICH) guideline.

To obtain the test samples, the fibroin freeze-dried powder (0.5 g) was hydrolyzed by 10 mL of 6 N HCl for 24 h in the nitrogenic atmosphere at 110 ± 5 °C, followed by pH adjustment to 1–3 by 50% w/v KOH. Then, the resulted amino acid solutions were reacted with Fmoc N-hydroxysuccinimide ester at 40 °C for 30 min to form the fluorescent derivatives. Finally, the samples were subjected to HPLC analysis and the percentages of each amino acid in fibroin molecules were calculated based on standard of the respective reference amino acids.

#### 2.4.3 Molecular weights

The molecular weights of the SFT and SFV were assessed by protein sodium dodecyl sulfate-polyacrylamide (SDS-PAGE) gel electrophoresis, following standardized protocol with minor modification [[22], [23], [24]]. Briefly, SFT/SFV (10 mg) was dissolved in 1 mL DI water to make fibroin solution. Then, 40 μL of these fibroin solution was mixed with 10 μL of 2× SDS solution composed of 0.125 M Tris-HCl pH 6.8, 4% SDS, 20% glycerol, 1% 2-mercaptoethanol, and 0.2% bromophenol blue. Finally, the mixtures were boiled for 10 min, and subjected on 8% polyacrylamide gel using the vertical electrophoresis machine (Mini PROTEAN Tetra Cell, Bio-Rad). The molecular mass (M) protein scale of 10–180 kDa (Thermo PageRuler Prestained Protein Ladder) was used as a reference.

#### 2.4.4 Molecular structures

The molecular and chemical structures of SFT and SFV were measured using standard analytical methods of Fourier-transform infrared spectroscopy (FTIR), X-ray diffraction (XRD), and differential scanning calorimetry (DSC).

##### 2.4.4.1 Fourier-transform infrared spectroscopy (FTIR)

The FTIR analysis of the SFV and SFT was performed on a NICOLET 6700 (Thermo) spectrophotometer (wavenumber ranging from 4000 to 500 cm^−1^) with KBr pellet method. The spectra, collected at 4.0 cm^−1^ resolution, were measured in a desiccated air purge at coaddition of 256 interferograms. The empty KBr pellet was used to determine background signals and subtracted from the sample reading.

##### 2.4.4.2 X-ray diffraction (XRD)

The XRD was measured on a D8 Advance XRD instrument (Bruker), using Cu Kα radiation generated at 45 kV and 36 mA (λ = 0.154 nm). The lyophilized SFV and SFT were spread out onto quartz substrates and analyzed from 5 to 50° (2θ) at scan speed of 2°/min.

##### 2.4.4.3 Differential scanning calorimetry (DSC)

The DSC analysis was conducted using DSC 200 F3 (Netzsch, Germany) equipped with a ceramic:FRS6 sensor, and proceeded at a temperature range of 0–300 °C and a scanning rate of 3 K/min, in nitrogen gas flow of 20 mL/min. The lyophilized SFV and SFT (5–8 mg) was subjected in an aluminum pan, and measured with a blank pan as reference.

#### 2.4.5 Crystallinity

The fibroin crystallinity was assessed by a value called crystallinity index (CI), defined as the crystalline fractions/ratios of a material/formulation and calculated by dividing the crystalline signal intensities by the sum of the crystalline and amorphous signals' intensities [3]. In this study, to calculate the CI, the FTIR signals’ intensities were utilized [3]. For this, the fibroin amide I and amide II bands were utilized to calculate the CI, following equations (1), (2).

(1) CI_IRI_ = D_1622_/ (D_1622_ + D_1646_)

(2) CI_IRII_ = D_1517_/ (D_1517_ + D_1560_)

where CI_IRI_, D_1622_, and D_1646_ are the CI values, the crystalline portions density, and the amorphous portions density of the amide I bands. Similarly, CI_IRII_, D_1517_, and D_1560_ are the CI values, the crystalline portions density, and the amorphous portions density of the amide II bands.

### 2.5 Particles characterization

The micro-/nanoparticles made from SFT and SFV were characterized and compared their properties in terms of size and zeta potential, morphology, structure and crystallinity, drug entrapment and release profile, biostability, and hematotoxicity.

#### 2.5.1 Size and zeta potential

The particles sizes and polydispersity indexes (PI) were determined by dynamic light scattering (DLS) method, whereas the zeta potentials were measured by phase analysis light scattering (PALS) method. The instrument was operated at a fixed wavelength of 632.8 nm with a BI-200SM Goniometer linked with a BI-9010AT digital correlator. The zeta potential values were calculated using the Smoluchowski equation.

#### 2.5.2 Morphology

The particle morphology was observed using field emission scanning electron microscopy (FESEM, S4800 Hitachi, Japan). The FNP-SFT and FNP-SFV lyophilized powders were re-dispersed in DI water, and the dispersions were dropped on a SEM stub with aluminum tape, followed by drying at ambient temperature, gold-coated, and subjected to FESEM analysis. The images were obtained using the software included in the machine.

#### 2.5.3 Structure and crystallinity

The structure and crystallinity of FNP-SFT and FNP-SFV were determined and calculated using the FTIR technique, in similar approach and experimentation with the respective fibroin properties (section 2.4.4 and 2.4.5).

#### 2.5.4 Drug entrapment efficiency

To determine the drug entrapment efficiency of the FNP-SFT and FNP-SFV, the indirect method was employed. To this end, the remaining AmB, after being loaded into the particles, was separated by centrifugation, and UV–Vis spectroscopic measured at 408 nm using a Jasco V730 spectrophotometer. The unloaded AmB amount was then determined based on AmB standard curve (y = 0.0151x - 0.0018, R^2^ = 0.9990), and the AmB entrapment efficiency was calculated using equation (3).

(3) $$\%\;AmB\;entrapment\;efficien\mathrm{cy}=\frac{Initial\;amount-Unloaded\;amount}{Initial\;amount}\times 100\%$$

#### 2.5.5 Drug release profile

The shake-flask technique was used to determine the release characteristics of AmB loaded FNP-SFT and FNP-SFV. The phosphate buffer pH 6.8 containing 10% w/v Tween 80 was used to establish the sink condition (i.e., the medium that could dissolve at least 3 times the AmB amount in the particles) [25]. The lyophilized particles were re-dispersed in 40 mL of the prepared media and agitated at 100 rpm with an orbital shaker at 37 °C for 360 min. An aliquot (1 mL) of the sample was withdrawn at a frequency of 30 min. To ensure a steady volume, the fresh medium was replenished immediately after sampling. The samples were then centrifuged at 18000 rpm for 10 min, and the released AmB in the supernatant was UV–Vis spectroscopic measured at 408 nm. The percentages of AmB release was calculated based on equation (4).

(4) $$\%\;AmB\;release=\frac{C_{t}V_{0}+V\sum_{1}^{t-1}C_{i}}{M_{0}-\sum_{1}^{t-1}M_{i}}\times 100\;\left(\%\right)$$

where C_t_, C_i_ are the concentrations of the released AmB at the time point t and i, V_0_ is the total volume of dissolution buffer (20 mL), V is the withdrawal sample volume at each time point (1 mL), M_0_ is the initial amount of AmB, and M_i_ is the total amount of withdrawal AmB at the time point i.

#### 2.5.6 Biostability

The biostability of FNP-SFT and FNP-SFV in the biological blood was determined using the weight-loss experiments [4]. Briefly, the particles (10 mg) were dispersed in 1 mL of the sheep whole blood and stirred at 200 rpm, 37 °C, for 12 h. At each pre-determined time point of 0, 3, 6, and 12 h, 1 mL of DI water was added to the samples to lyse the blood, followed by centrifugation at 18000 rpm for 5 min. The precipitated solid were thoroughly washed thrice with DI water by centrifugation techniques. Finally, the precipitates were dried at 60 °C until constant weight, and the remaining particle weights were measured by an analytical balance. The particle biostability was determined based on the percentage of particle weight loss following equation (5). The blood samples with no particles were utilized as controls.

(5) $$\%\;Weight\;loss=\frac{Initial\;amount\;\left(10\;mg\right)-\left(Remaining\;amount-Control\right)}{Initial\;amount\;\left(10\;mg\right)}\times 100\%$$

#### 2.5.7 Hematotoxicity

The hematotoxicity, determined by the hemolysis percentage, of the FNP-SFT and FNP-SFV was in-vitro investigated on the sheep red blood cells [5,8]. To this end, the sheep erythrocytes were obtained by centrifugation the sheep whole blood at 4000 rpm for 5 min, washed thrice with PBS, and diluted in PBS at a concentration of 1% hematocrit (1% w/v). Next, the FNP-SFT and FNP-SFV, at various amounts, were incubated with 1 mL of the prepared red blood cells at 37 °C for 30 min. Finally, the samples were centrifuged at 4000 rpm for 5 min, and the hemoglobin in the supernatants was UV–Vis spectroscopic measured at 540 nm. The hemolysis percentages were then calculated using equation (6).

(6) $$\%\;Hemolysis=\frac{At-An}{Ap-An}\times 100\%$$

where At, Ap, An are the absorbance values of the test samples, the positive control (DI water), and the negative control (PBS), respectively.

### 2.6 Statistical analysis

All experiments, both qualitative and quantitative, were conducted in at least three times. The comparisons between samples, if any, were performed by the analysis of variance (ANOVA) tests, with p values of less than 0.05 for significant associations.

## 3 Results and discussions

### 3.1 Comparisons between SFT and SFV

#### 3.1.1 Physical properties

**Fig. 2:**
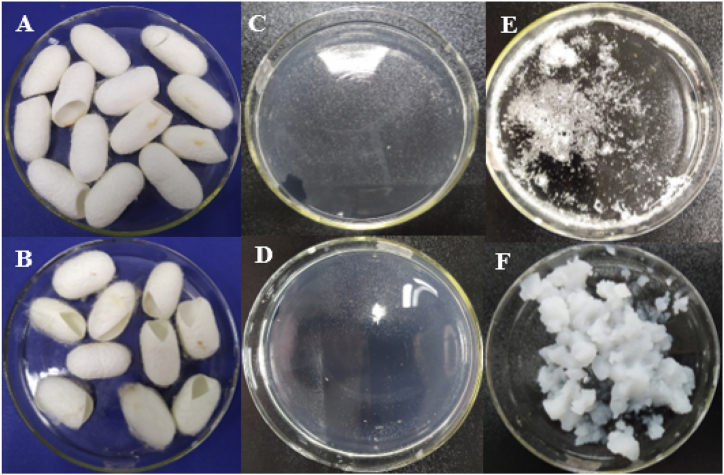
Physical appearances of (A) Thai dried silkworm cocoons, (B) Vietnamese dried silkworm cocoons, (C) Thai silk fibroin (SFT) aqueous solution (initial time, t = 0), (D) SFT aqueous solution after 1-month storage at 4 °C (t = 30 days), (E) Vietnamese silk fibroin (SFV) aqueous solution (initial time, t = 0), and (F) SFV aqueous solution after 1-month storage at 4 °C (t = 30 days).

The fibroin amounts extracted from the two varieties of silk cocoons were significantly different. Utilizing the same extraction procedure, the extraction efficiencies for Thai silk and Vietnamese silk were 58.65 ± 3.12% and 20.08 ± 2.47%, respectively. Since different silk types with distinct silkworm living environments and cultivation conditions yield significantly different fibroin and sericin content [26], our results suggest that the Thai silkworm might produce silk with more fibroin content than that of the Vietnamese silkworm (Fig. 2A and B). Additionally, after one month storage at 4 °C, the extracted SFT solution did not precipitate, whereas the SFV solution demonstrated clear fibroin precipitations (Fig. 2C-F). This finding revealed that fibroin from Thailand silk was more physically stable than fibroin from Vietnamese silk, which could be due to the inherent large molecular weight and high crystallinity of the latter (discussed in the next sections). Thus, the water-soluble silk-I amorphous structure of SFV was rapidly transformed into the water-insoluble silk-II crystalline structure, and possibly, silk-III structure that could be observed at the fibroin solution's gas-liquid interface [1].

#### 3.1.2 Amino acid compositions

The amino acid compositions of SFT and SFV are presented in Table 1. No significant differences, in both the amino acid types and amounts, were noted between these two silk fibroins. Additionally, the data were in well accordance with the literature, of which the glycine, alanine, and serine were the three most abundant amino acids in fibroin (accounted for ∼45%, ∼25%, and ∼10% w/w, respectively) [27,28]. Therefore, the silkworm living environments and cultivation conditions might not strongly affect the fibroin chemical contents.

**Table 1:** Amino acid compositions of Thai silk fibroin (SFT) and Vietnamese silk fibroin (SFV).

Amino acid	Percentage (% w/w)	
	Thai silk fibroin	Vietnamese silk fibroin
Glycine	46.1	46.9
Alanine	25.8	25.4
Serine	9.8	9.5
Tyrosine	5.7	6.2
Valine	2.3	2.0
Aspartic acid	1.6	1.5
Glutamic acid	1.3	1.1
Threonine	0.9	0.8
Arginine	0.6	0.5
Isoleucine	0.6	0.5
Leucine	0.5	0.6
Phenylalanine	1.0	0.9
Proline	0.7	0.7
Lysine	0.6	0.6
Others	2.5	2.8

#### 3.1.3 Molecular weights

**Fig. 3:**
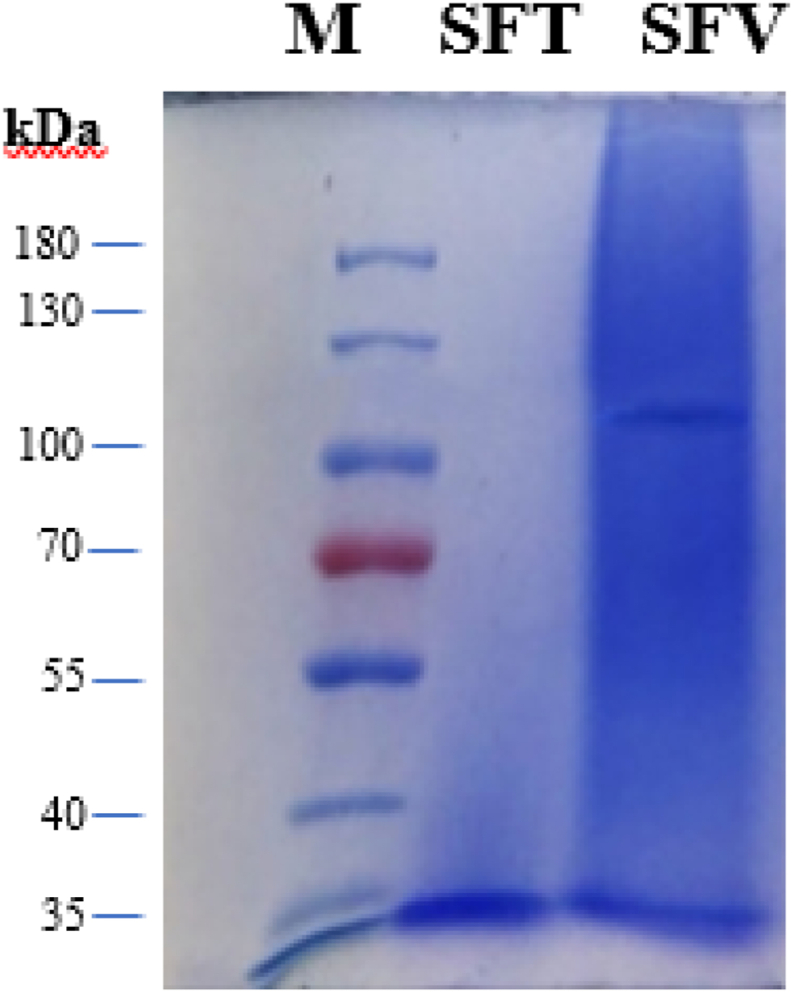
Sodium dodecyl sulfate-polyacrylamide gel electrophoresis (SDS-PAGE) of Thai silk fibroin (SFT) and Vietnamese silk fibroin (SFV). M: marker. The full, non-adjusted gel image is presented in Figure S1.

Generally, the fibroin molecule composes of a heavy chain (∼390 kDa) and a light chain (∼26 kDa) joined by a disulfide bridge, and non-covalently bound by a P-25 glycoprotein [3]. Nevertheless, the fibroin weights are affected by various factors of extraction process, silkworm varieties, and cultivation conditions [29,30]. For instance, during the sericin-removal process with Na_2_CO_3_, the salt alkaline nature might lower the macromolecules weights in the peptide chain, thus influencing the weight of the produced fibroin [24]. In this study, besides the differences in the Thai and Vietnamese silkworms, all other factors were kept the same. Thus, the discrepancy in molecular weights between SFT and SFV (Fig. 3) was solely due to the different silkworm types. Specifically, both SFT and SFV samples show a clear band at around 30 kDa, which correlates to the presence of the light chains and P-25 proteins that were cleaved from both ends of the fibroin chains [30]. Interestingly, the SFV possessed a weak band at approximately 115 kDa, indicating the fragmented heavy chain presence in the sample. On the other hand, this band was absent in the SFT sample, suggesting that most of the SFT molecules were being hydrolyzed and divided into small-length and low-molecular-weight polypeptide chains during the extraction process. Conclusively, the SFV structures contained both heavy and light chains, and P-25 proteins, whereas the SFT mostly consisted of light chains and P-25 proteins.

#### 3.1.4 Molecular structures and crystallinity

**Fig. 4:**
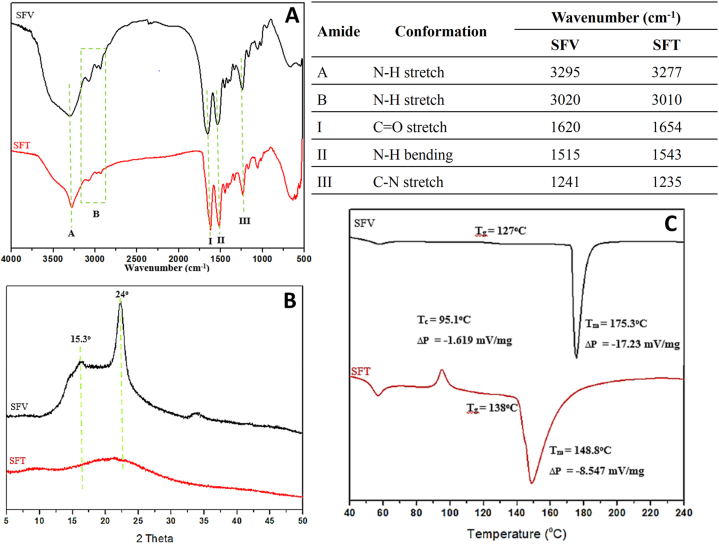
Analytical spectra of Thai silk fibroin (SFT) and Vietnamese silk fibroin (SFV). (A) Fourier-transform infrared spectroscopy (FTIR) and the corresponding peak table, (B) X-ray diffractometry (XRD), and (C) Differential scanning calorimetry (DSC).

To elucidate the molecular structures of SFT and SFV, analytical methods of FTIR, XRD, and DSC were employed. Firstly, the FTIR spectra of both fibroin demonstrated characteristic fibroin peaks consisting of five main amide bands (A, B, I, II, and III) (Fig. 4A). The conformations of these peaks are also presented in Fig. 4. Notably, compared to those of the SFT, the signals of amide I, II, and III of the SFV were shifted to a different wavenumber of 1620 cm^−1^, 1515 cm^−1^, and 1241 cm^−1^, respectively. These peak locations have been confirmed to be the crystalline portions of the fibroin water-insoluble silk-II polymorph [3]. Therefore, the SFV structure was mainly the crystalline silk II, whereas the SFT structure was mainly the amorphous silk I. Secondly, in agreement with the FTIR results, the XRD also shows the SFV sharp crystalline peak at the 2θ of 24° [3], while this peak signal of the SFT was broad and insignificant (Fig. 4B). Finally, regarding the DSC thermographs (Fig. 4C), both the SFT and SFV possess the endothermic peaks in the temperature range of 60–80 °C, indicating the dehydration process of both SFT and SFV samples [31]. Interestingly, the SFT exhibits an exothermic peak at 95.1 °C, suggesting a temperature-assisted conformational alteration from silk I to silk II polymorph [3,31]. This peak is absent in the SFV, which re-confirms that most of its structure was already in the crystalline silk II. Additionally, the endothermic peaks at high temperatures of 148.8 °C for SFT and 175.3 °C for SFV, which are attributed to the fibroin decomposition due to the changes of the random-sheet coils, indicate that SFV is more thermal stable than SFT [32]. Conclusively, all three analytical techniques of FTIR, XRD, and DSC confirmed that SFV was mainly stayed in the crystalline silk-II polymorph, whereas most of the SFT structure was amorphous silk I.

Since fibroin is a semi-crystalline material composed of both crystalline and amorphous regions, its crystallinity could be quantitatively calculated based on the FTIR spectra [3]. To this end, the CI values for both the amide-I and amide-II peaks of SFV were higher than those of SFT (0.50 vs. 0.40 for amide I, and 0.43 vs. 0.35 for amide II), which, again, indicated that the SFV was more crystalline than the SFT.

In summary, taking all data into consideration, it could be concluded that both SFT and SFV possessed similar amino acid compositions, indicating that the geographical and cultivation conditions did not affect much on the fibroin chemical contents. On the other hand, the fibroin structures were significantly different between the two silk types. The SFV structures contained both heavy and light chains, and P-25 proteins, thus mainly possessing a crystalline silk-II polymorph. Conversely, the SFT structure mostly composed of light chains and P-25 proteins, thus mainly having the amorphous silk-I polymorph. In the next section, the FNP formulated from these two distinct fibroin were thoroughly investigated and compared.

### 3.2 Comparisons between FNP-SFT and FNP-SFV

#### 3.2.1 Size, morphology, and zeta potential

**Fig. 5:**
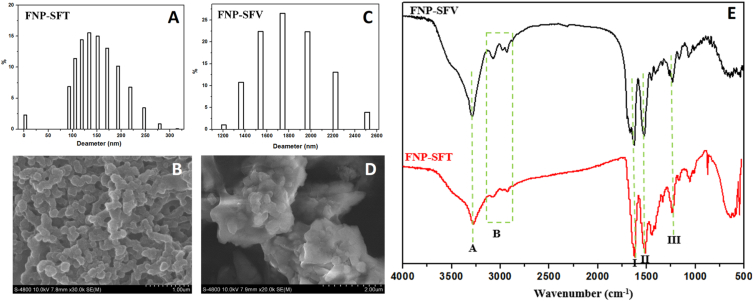
(A and C) Particle sizes and size distributions of the fibroin micro-/nanoparticles fabricated from Thai silk fibroin (FNP-SFT) and Vietnamese silk fibroin (FNP-SFV), and their corresponding scanning electron microscopic (SEM) images (B and D, scale bars of 1 μm and 2 μm, respectively). The particles Fourier-transform infrared (FTIR) spectra are also demonstrated (E).

Utilizing the same preparation method, the FNP were fabricated from SFT and SFV, and characterized and compared their properties. In terms of the particle sizes, the DLS analysis revealed that the particles produced from SFT are, however, many orders of magnitude smaller (i.e., in the nano-ranged sizes of 100–200 nm) than those prepared from SFV, which possessed a micro-ranged sizes of 1200–2500 nm (Fig. 5A and C). Additionally, the FNP-SFV demonstrated a much broader size distribution than FNP-SFT. This could be due to the fact that compared to the amorphous silk-I structure of SFT, SFV had a crystalline structure, thus, during the desolvation process, the SFV molecules rapidly aggregated and produced bigger-and-non-homogeneous particles than those of the SFT [5,8]. Furthermore, the SEM micrographs re-confirmed this phenomenon, as the FNP-SFT particles were nano-sized uniform spheres/pellets, whereas the FNP-SFV sample had irregular shape with numerous folds and smaller particles attached on their surfaces (Fig. 5B and D). Moreover, the FNP-SFT demonstrated aggregated particles, in well agreement with the previous reports [33,34]. Regarding the zeta potentials, the FNP-SFT and FNP-SFV had comparable negative charge of −25 mV, indicating the inherent charge of fibroin [14].

#### 3.2.2 Structure and crystallinity

Fig. 5E depicts the FTIR spectra of FNP-SFT and FNP-SFV particles, which exhibit the emergences of fibroin-specific peaks (i.e., amide A, B, I, II, and III) identical to those of the SFT and SFV samples (Fig. 4A). This demonstrates that the particles could maintain the characteristics of fibroin, without the effects of formulation processes. Furthermore, due to the formations of cross-links during the fibroin self-granulation, which produced more anti-parallel β-sheet structures, the crystallinity of the FNP was higher than that of the raw fibroin [3,6]. Accordingly, the CI values of the FNP-SFV was also greater than that of FNP-SFT (0.52 vs. 0.49 for amide I, and 0.50 vs. 0.47 for amide II), which are perfectly compatible with the raw fibroin crystallinity data.

#### 3.2.3 Drug entrapment efficiency and release profile

The ability to encapsulate and release the loaded drugs of FNP-SFT and FNP-SFV were further investigated, using the model drug AmB. To this end, the FNP-SFT and FNP-SFV possessed a drug EE% of 79.3 ± 5.1% and 64.3 ± 4.4%, respectively. This could be due to the high crystallinity of the FNP-SFV particles, with high β-sheet structure, consequently restricting the drug's entry into the particles and lowering the loading efficiency. Nevertheless, our results were different than those of the previous reports, which indicated that FNP with higher crystallinity showed better EE% than that of the particles with lower crystallinity, using the model drug of alpha mangostin and paclitaxel [5,8]. Therefore, in-depth investigations are necessary to elucidate these controversies.

Regarding the AmB release profiles from the particles. Although the experiments were performed in the sink condition, in which the AmB solubility was not a factor affecting its release rate, no significant AmB amount was released from both particles (<4%). Our results were in agreement with the previous report that encapsulating the AmB in the FNP [9]. This might be because AmB binds strongly to the fibroin molecules, possibly via chemical interactions such as hydrogen bonding and hydrophobic interactions, consequently hinder the AmB release rates.

#### 3.2.4 Biostability and hematotoxicity

**Fig. 6:**
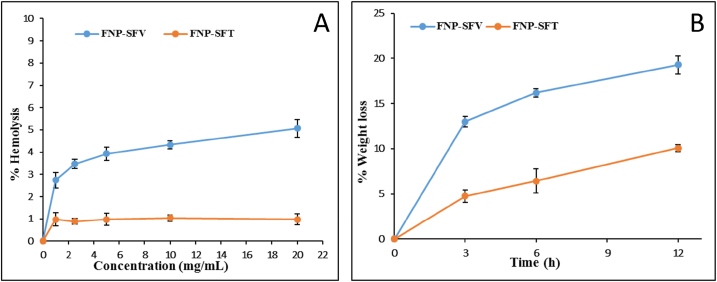
(A) Hemolysis action and (B) biostability profile, in terms of % weight loss, in the sheep blood, of fibroin micro-/nanoparticles fabricated from Thai silk fibroin (FNP-SFT) and Vietnamese silk fibroin (FNP-SFV).

The fibroin structure, as well as its crystallinity, have been proven to significantly affect the FNP bio-properties [[3], [4], [5],^8^]. Thus, we investigated two important bio-properties, the stability in the whole blood (i.e., biostability) and the ability to lyse the red blood cells of FNP-SFT and FNP-SFV, using the sheep blood. For this, the FNP-SFV possessed significantly higher hemolysis action (Fig. 6A) and lower biostability (i.e., higher weight loss) (Fig. 6B), than those of the FNP-SFT. Because the FNP-SFV particles have a high crystallinity, the impact of the particles and the red blood cells during the shaking experiment can provide a bigger force than that of the FNP-SFT particles, consequently disrupting the structure of erythrocytes. Furthermore, the FNP-SFV big particle size (i.e., micron) and their rough surfaces could compress and squash the fragile erythrocytes in the medium. Nevertheless, both types of particles, at concentrations of as high as 20 mg/mL, only lysed less than 5% of the total red blood cells, indicating that the FNP are safe to the blood.

Regarding the biostability, the FNP-SFV degraded three times faster than FNP-SFT in the first 3 h, and this rate steadily dropped with time. The quick breakdown of FNP-SFV particles could be explained by their large particle size and porous shape, making the exterior bonds become weaker, thus allowing the blood proteolytic enzymes to easily come into contact and break down the fibroin molecules.

## 4 Conclusions

**Fig. 7:**
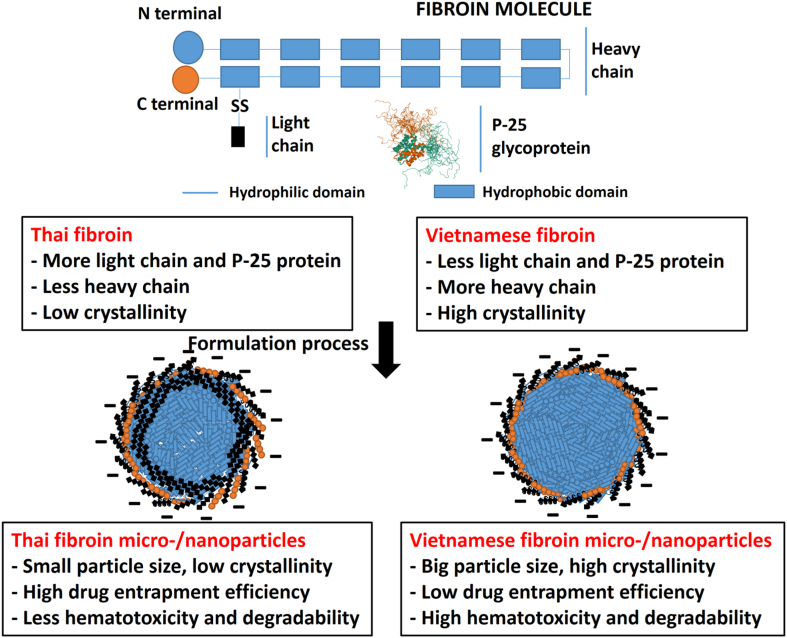
Schematic comparisons on the properties between the Thai silk fibroin (SFT) and Vietnamese silk fibroin (SFV), and their corresponding micro-/nanoparticles.

The study discovered significant variations in the characteristics of Thai silk fibroin and Vietnamese silk fibroin, as well as their corresponding fibroin micro-/nanoparticles. The main differences between these two types of silk fibroin, and micro-/nanoparticles, are summarized in Fig. 7. The exceptional qualities of Thai silk fibroin, such as stability and the capacity to generate fine nanoparticles, outperform those of Vietnamese silk fibroin. However, the crystallinity and thermal stability of fibroin derived from Vietnamese silk are significantly higher. These distinctions serve as the foundation for appropriate applications of each fibroin kind to best promote its qualities and usefulness.

## Author contribution statement

Ngoc Yen Nguyen: Conceived and designed the experiments; Performed the experiments; Analyzed and interpreted the data; Wrote the paper.

Thi Ngoc Phuong Nguyen: Performed the experiments; Analyzed and interpreted the data; Wrote the paper.

Nguyen Ngoc Huyen, Van De Tran & Tran Thi Bich Quyen: Performed the experiments; Wrote the paper.

Huynh Vu Thanh Luong: Conceived and designed the experiments; Analyzed and interpreted the data; Contributed reagents, materials, analysis tools or data; Wrote the paper.

Duy Toan Pham: Conceived and designed the experiments; Contributed reagents, materials, analysis tools or data; Wrote the paper.

## Data availability statement

Data will be made available on request.

## Funding

This research is funded by the Vietnam 10.13039/501100005645Ministry of Education and Training under grant number B2022-TCT-04.

## Declaration of competing interest

The authors declare that they have no known competing financial interests or personal relationships that could have appeared to influence the work reported in this paper.
