# Supplementary material for: Onto the differences in formulating micro-/nanoparticulate drug delivery system from Thai silk and Vietnamese silk: A critical comparison
Source: Heliyon. 2023 Jun 2;9(6):e16966. doi: 10.1016/j.heliyon.2023.e16966 (PMC10361021; doi:10.1016/j.heliyon.2023.e16966)
Supplement: Multimedia component 1 [file mmc1.docx]

**
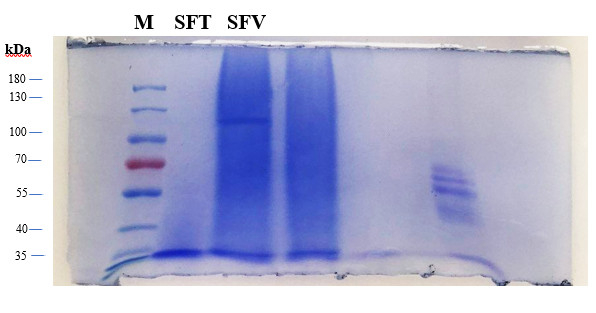
**

**Figure S1.** The full, non-adjusted SDS-PAGE gel image of Thai silk fibroin (SFT) and Vietnamese silk fibroin (SFV). M: marker.
